# Role of Transmembrane Proteins for Phase Separation and Domain Registration in Asymmetric Lipid Bilayers

**DOI:** 10.3390/biom9080303

**Published:** 2019-07-25

**Authors:** Guilherme Volpe Bossa, Sean Gunderson, Rachel Downing, Sylvio May

**Affiliations:** 1 Department of Physics, Institute of Biosciences, Humanities and Exact Sciences, São Paulo State University (UNESP), São José do Rio Preto, SP 15054-000, Brazil; 2 Department of Physics, North Dakota State University, Fargo, ND 58108-6050, USA

**Keywords:** biomembrane, lipid bilayer, phase separation, inter-leaflet coupling, membrane domain

## Abstract

It is well known that the formation and spatial correlation of lipid domains in the two apposed leaflets of a bilayer are influenced by weak lipid–lipid interactions across the bilayer’s midplane. Transmembrane proteins span through both leaflets and thus offer an alternative domain coupling mechanism. Using a mean-field approximation of a simple bilayer-type lattice model, with two two-dimensional lattices stacked one on top of the other, we explore the role of this “structural” inter-leaflet coupling for the ability of a lipid membrane to phase separate and form spatially correlated domains. We present calculated phase diagrams for various effective lipid–lipid and lipid–protein interaction strengths in membranes that contain a binary lipid mixture in each leaflet plus a small amount of added transmembrane proteins. The influence of the transmembrane nature of the proteins is assessed by a comparison with “peripheral” proteins, which result from the separation of one single integral protein into two independent units that are no longer structurally connected across the bilayer. We demonstrate that the ability of membrane-spanning proteins to facilitate domain formation requires sufficiently strong lipid–protein interactions. Weak lipid–protein interactions generally tend to inhibit phase separation in a similar manner for transmembrane as for peripheral proteins.

## 1. Introduction

Lipids in membranes tend to mix nonideally [1]. Many lipid mixtures are known for their ability to phase separate or form domains [2]. Of special interest is domain formation in biomembranes because of its putative functional role associated with the membrane raft hypothesis [3,4,5]. The plasma membrane of mammalian cells is asymmetric and multicomponent, but its lipid composition has often been described—in a first-order approximation—as consisting of phosphatidylcholine (PC) and sphingomyelin (SM) in the outer leaflet, phosphatidylserine (PS) and phosphatidylethanolamine (PE) in the inner leaflet, and cholesterol as being able to populate both leaflets [6]. It is well known from experiments in model membranes that the lipids in the outer leaflet appear to represent a mixture of saturated and unsaturated lipids with cholesterol that forms liquid-ordered (*lo*) domains [7,8]. No such tendency is observed for the lipids in the inner leaflet [9]. However, there are some hints—concluded mostly from computer simulations [10,11]—that suggest *lo* domains could also exist in the inner leaflet of the plasma membrane and that they are spatially registered with those in the outer leaflet [6].

The raft hypothesis remains controversial [12,13], but it has sparked a large number of experimental [14,15], computational [16], and theoretical [17] studies about domain formation in model membranes, with an increasing focus on inter-leaflet domain coupling in asymmetric bilayers [7,18,19,20]. Despite being in their fluid state, sufficiently large domains located in the apposed leaflets of a lipid bilayer tend to register due to a domain mismatch energy on the order of $0.1-0.2\;k_{B}T/{\mathrm{nm}}^{2}$ [21] ($k_{B}T$ is the thermal energy unit: Boltzmann constant times absolute temperature). There is experimental evidence that the mismatch energy is large enough to not only register preexisting domains, but to even induce domains in one leaflet by an existing domain in the apposed leaflet [22]. The origin of the mismatch energy has been suggested to be mostly entropic [23,24], stemming from a more efficient dynamic penetration of the bilayer’s midplane by the lipid tails in the registered as compared to the unregistered domain arrangement. Sufficiently small domains may antiregister to minimize the line tension by hydrophobic domain matching [25,26]. Recent theoretical modeling on the mean-field level of a lattice gas has addressed the calculations of phase diagrams in asymmetric membranes [25,27,28,29,30,31,32]. Here, the domain mismatch energy penalty drives domain registration, but domain formation itself is driven foremost by interactions of the lipids in the same leaflet. This can lead to a rich phase behavior depending on the lipid–lipid interaction strength within each leaflet and the strength of the inter-leaflet domain coupling.

As mentioned, lipid domains can be coupled across the membrane “thermodynamically” through a domain mismatch energy. Here, registered domains are energetically (but not structurally) connected across the bilayer. There is another possibility that has been suggested [33,34,35] but not further pursued: transmembrane proteins or peptides, or membrane-spanning lipids (such as bolalipids [36]), provide a “structural” domain coupling mechanism that may act in conjunction with the above-mentioned thermodynamic mechanism of energy penalties for mismatching domains. Obviously, membrane-spanning proteins are able to physically connect the domains they are associated with across the membrane, irrespective of the inter-leaflet domain interaction energy. In addition, one single transmembrane protein has a lower in-plane translational entropy in a membrane as compared to two equivalent “peripheral” proteins that result from the separation of the transmembrane protein into two independent units. The lower entropy too is expected to favor domain registration. On the other hand, transmembrane proteins of different hydrophobic lengths invoke hydrophobic mismatch penalties in membranes [37] that will affect their ability to induce phase separation. This was observed, for example, by Ackerman and Feigenson [38] in a coarse-grained molecular dynamic simulation of a four-component lipid membrane in the presence of additional transmembrane WALP peptides of varying lengths. Independently of their length, however, all WALP peptides were observed to increase domain alignment. The structural coupling mechanism is not confined to transmembrane proteins; it also applies to bolalipids [39] and even to lipids with long tails such as monosialotetrahexosylganglioside (GM1) [40] and other long saturated acyl chains [11] that interact with the lipids in the apposed leaflet.

The objective of the present work is to propose and analyze a minimal model for phase separation in a mixed lipid bilayer that is subject to the two distinct inter-leaflet coupling mechanisms: a thermodynamic one due to the presence of a compositional mismatch between the two leaflets and a structural one due to the presence of transmembrane proteins. The term “transmembrane protein” stands as a representative for any type of membrane-spanning molecule that is able to interact with the lipids in both leaflets, including integral proteins, transmembrane peptides, bolalipids, and even long-chain lipids. We propose a bilayer-type lattice model, with two two-dimensional lattices stacked one on top of the other. Lipids of two different types (referred to as A and B) occupy one lattice site each, whereas transmembrane proteins consist of two lattice sites that span the bilayer. Hence, each leaflet contains a ternary mixture consisting of two different lipid types and the protein. We introduce all relevant lipid–lipid and lipid–protein interactions and analyze the model on the mean-field level by calculating spinodal surfaces, critical points, tri-critical points, as well as coexistence regions and tie lines in some cases. We demonstrate the ability of transmembrane proteins to facilitate phase transition and to register domains across the bilayer. Our work represents a first attempt to approach an understanding of the three-dimensional phase diagram of a mixed protein-containing bilayer—the richness of the features in the phase diagram justifies the simplicity of our lattice approach, including the neglect of effects due to hydrophobic mismatch, membrane bending, and multi-body interactions.

## 2. Theory

**Figure 1:**
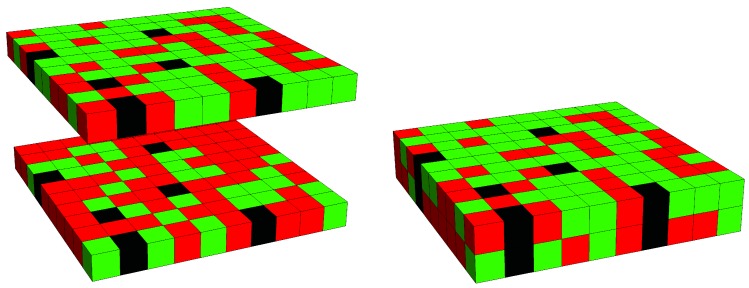
Lattice model for a mixed lipid bilayer that contains transmembrane proteins. The snapshot shows the two membrane leaflets separated (left diagram) and merged into a bilayer (right diagram). Positions of lipids of type A (green) and lipids of type B (red) are uncorrelated across the lattice. Number and locations of transmembrane protein segments (black) are identical in the two leaflets. Note that both leaflets may have different lipid compositions. The displayed snapshot corresponds to a square lattice (z=4) of size M=10×10 and mole fractions $\phi =A_{ext}/M=0.62$ (the upper leaflet), $\psi =A_{int}/M=0.30$ (the lower leaflet) and α=P/M=0.06.

We consider two-dimensional lattice models for the external (“*ext*”) and internal (“*int*”) leaflets of a lipid bilayer that contains a fixed number of transmembrane proteins. The two lattices have the same coordination number *z* (for example, z=4 for a cubic and z=6 for a hexagonal lattice) and reside on top of each other so that each lattice site on the external lattice contacts exactly one lattice site on the internal lattice. Each lattice has a total of *M* lattice sites; the external one hosts *P* transmembrane proteins, $A_{ext}$ lipids of type A, and $B_{ext}=M-P-A_{ext}$ lipids of type B. Similarly, the internal lattice hosts *P* transmembrane proteins, $A_{int}$ lipids of type A, and $B_{int}=M-P-A_{int}$ lipids of type B. Because transmembrane proteins span the entire bilayer, the protein positions in each leaflet are exactly the same for each microstate. The illustration of one specific microstate in Figure 1 shows the correlations of protein numbers and positions across the two lattices.

### 2.1. Free Energy of a Lipid Membrane That Contains Transmembrane Proteins

We consider a mean-field Helmholtz free energy F=U−TS of our lattice model at fixed temperature *T*. Its internal energy $U=U_{ext}+U_{int}+U_{coupl}$ reflects nearest-neighbor interactions within each lattice ($U_{ext}$ and $U_{int}$) and a coupling term across the two lattices ($U_{coupl}$). The entropy $S=k_{B}\ln\Omega$ (with $k_{B}$ denoting Boltzmann’s constant) accounts for the number of available states,

(1) $$\Omega =\frac{M!\;(M-P)!}{P!\;A_{ext}!\;A_{int}!\;B_{ext}!\;B_{int}!}.$$

On the level of the random mixing approximation [41,42], the in-plane nearest-neighbor interaction energies $U_{ext}$ in the external layer and $U_{int}$ in the internal layer can be expressed as

(2) $$\frac{U_{ext}}{k_{B}T}=\chi_{L}^{ext}\frac{B_{ext}A_{ext}}{M}+\chi_{P}^{ext}\frac{PA_{ext}}{M}+{\bar{\chi }}_{P}^{ext}\frac{PB_{ext}}{M},\;\frac{U_{int}}{k_{B}T}=\chi_{L}^{int}\frac{B_{int}A_{int}}{M}+\chi_{P}^{int}\frac{PA_{int}}{M}+{\bar{\chi }}_{P}^{int}\frac{PB_{int}}{M},$$

where

(3) $$\begin{array}{ccc} \chi_{L}^{ext} & = & \frac{z}{2}\;\left[\omega_{AB}^{ext}-\frac{1}{2}\left(\omega_{AA}^{ext}+\omega_{BB}^{ext}\right)\right],\;\chi_{L}^{int}=\frac{z}{2}\;\left[\omega_{AB}^{int}-\frac{1}{2}\left(\omega_{AA}^{int}+\omega_{BB}^{int}\right)\right],\\ \chi_{P}^{ext} & = & \frac{z}{2}\;\left[\omega_{AP}^{ext}-\frac{1}{2}\left(\omega_{AA}^{ext}+\omega_{PP}^{ext}\right)\right],\;\chi_{P}^{int}=\frac{z}{2}\;\left[\omega_{AP}^{int}-\frac{1}{2}\left(\omega_{AA}^{int}+\omega_{PP}^{int}\right)\right],\\ {\bar{\chi }}_{P}^{ext} & = & \frac{z}{2}\;\left[\omega_{BP}^{ext}-\frac{1}{2}\left(\omega_{BB}^{ext}+\omega_{PP}^{ext}\right)\right],\;{\bar{\chi }}_{P}^{int}=\frac{z}{2}\;\left[\omega_{BP}^{int}-\frac{1}{2}\left(\omega_{BB}^{int}+\omega_{PP}^{int}\right)\right], \end{array}$$

are effective lipid A–lipid B, lipid A–protein, and lipid B–protein interaction strengths in the external and internal leaflets. These reflect the actual interaction strengths between lipid A-lipid A ($\omega_{AA}^{ext}$ and $\omega_{AA}^{int}$), lipid A–lipid B ($\omega_{AB}^{ext}$ and $\omega_{AB}^{int}$), lipid B-lipid B ($\omega_{BB}^{ext}$ and $\omega_{BB}^{int}$), lipid A–protein ($\omega_{AP}^{ext}$ and $\omega_{AP}^{int}$), lipid B-protein ($\omega_{BP}^{ext}$ and $\omega_{BP}^{int}$), and protein–protein ($\omega_{PP}^{ext}$ and $\omega_{PP}^{int}$), all expressed in units of $k_{B}T$.

The physical situation we are interested in is the presence of lipid A–lipid B interactions in each leaflet and preferential interactions of the proteins with only one lipid type. To this end, we may simply assume $\omega_{BP}^{ext}=\omega_{AA}^{ext}=\omega_{BB}^{ext}=\omega_{PP}^{ext}=0$ for the external leaflet and $\omega_{BP}^{int}=\omega_{AA}^{int}=\omega_{BB}^{int}=\omega_{PP}^{int}=0$ for the internal leaflet. This would leave us with $\chi_{L}^{ext}=z\omega_{AB}^{ext}/2$, $\chi_{P}^{ext}=z\omega_{AP}^{ext}/2$, $\chi_{L}^{int}=z\omega_{AB}^{int}/2$, and $\chi_{P}^{int}=z\omega_{AP}^{int}/2$, whereas ${\bar{\chi }}_{P}^{ext}$ and ${\bar{\chi }}_{P}^{int}$ both vanish. More generally, we assume everywhere in the present work ${\bar{\chi }}_{P}^{ext}={\bar{\chi }}_{P}^{int}=0$, whereas $\chi_{L}^{ext}$, $\chi_{P}^{ext}$, $\chi_{L}^{int}$, $\chi_{P}^{int}$ may all be non-vanishing. If the interactions are symmetric across the bilayer, we are left with only two interaction strengths that we refer to as $\chi_{L}=\chi_{L}^{ext}=\chi_{L}^{int}$ and $\chi_{P}=\chi_{P}^{ext}=\chi_{P}^{int}$. We also consider cases of asymmetric interactions where $\chi_{L}^{ext}\neq \chi_{L}^{int}$ or $\chi_{P}^{ext}\neq \chi_{P}^{int}$. The case $\chi_{L}^{ext}\neq \chi_{L}^{int}$ accounts for the different propensities of the lipids in the two leaflets of a plasma membrane to undergo phase separation [9].

Symmetry demands the lowest order term of the inter-leaflet coupling energy across the membrane to be quadratic; on the basis of the random mixing approximation, we obtain $U_{coupl}/k_{B}T=\Lambda \left(M-P\right){\left[\left(A_{ext}-A_{int}\right)/M\right]}^{2}$, where the coupling constant

(4) $$\Lambda =\frac{1}{2}\left[{\tilde{\omega }}_{AB}-\frac{1}{2}\left({\tilde{\omega }}_{AA}+{\tilde{\omega }}_{BB}\right)\right],$$

reflects the inter-leaflet interaction of lipid A with lipid B (${\tilde{\omega }}_{AB}$), of lipid A with lipid A (${\tilde{\omega }}_{AA}$), and of lipid B with lipid B (${\tilde{\omega }}_{BB}$). It is convenient to define the three mole fractions

(5) $$\phi =\frac{A_{ext}}{M},\;\psi =\frac{A_{int}}{M},\;\alpha =\frac{P}{M}.$$

Using Stirling’s approximation ln(x!)≈xlnx−x in the expressions for *S* as well as the definitions in Equation (5), we find for the total Helmholtz free energy $f=F/(Mk_{B}T)$, in units of $k_{B}T$ and per lattice site,

(6) $$\begin{array}{ccc} f & = & \alpha \ln\alpha -(1-\alpha )\ln(1-\alpha )+\phi \ln\phi +\psi \ln\psi \\ & + & \left(1-\alpha -\phi )\ln(1-\alpha -\phi )+(1-\alpha -\psi )\ln(1-\alpha -\psi \right)\\ & + & \chi_{L}^{ext}\phi \left(1-\alpha -\phi \right)+\chi_{L}^{int}\psi \left(1-\alpha -\psi \right)+\left(1-\alpha \right)\Lambda {\left(\phi -\psi \right)}^{2}+\chi_{P}^{ext}\alpha \phi +\chi_{P}^{int}\alpha \psi , \end{array}$$

where we again note that we assume ${\bar{\chi }}_{P}^{ext}={\bar{\chi }}_{P}^{int}=0$. Without that assumption, the free energy in Equation (6) would contain the additional contribution ${\bar{\chi }}_{P}^{ext}\alpha \left(1-\alpha -\phi \right)+{\bar{\chi }}_{P}^{int}\alpha \left(1-\alpha -\psi \right)$.

A specific goal of the present work is to quantify the phase behavior that follows from the function f=f(ϕ,ψ,α) specified in Equation (6), subject to fixing the interaction strengths $\chi_{L}^{ext}$, $\chi_{L}^{int}$, $\chi_{P}^{ext}$, $\chi_{P}^{int}$, Λ in the thermodynamic limits of fixed temperature *T* and an infinitely large membrane size, M→∞. The compositional variables ϕ, ψ, and α can vary independently within the ranges 0≤α≤1, 0≤ϕ≤1−α, and 0≤ψ≤1−α. However, the interesting protein mole fraction—on which we focus in the present work—is that of small α. We generally consider 0≤α<0.05. This seems reasonable because only the most protein-rich biological membranes such as the purple membrane of *Halobacterium halobium* have protein-to-lipid molar ratios of less than 1:50 [43]. Larger mole fractions may occur locally [44], but their consideration would not only add another layer of complexity to the present work; they would also raise concerns about the structural stability of membranes that the present simple lattice model is not designed to address.

The mole fractions ϕ, ψ, and α constitute three independent degrees of freedom. This renders the phase diagram three-dimensional, with a maximum of four phases that can coexist. We note two symmetries. The first one, f(ϕ,ψ,0)=f(1−ϕ,1−ψ,0), applies to a lipid bilayer that does not contain proteins. The second one, f(ϕ,ψ,α)=f(ψ,ϕ,α), is valid for a membrane with symmetric interactions, $\chi_{L}^{ext}=\chi_{L}^{int}$ and $\chi_{P}^{ext}=\chi_{P}^{int}$.

### 2.2. Spinodals and Critical Points

To investigate the phase behavior, we first consider the spinodal surface, which can be calculated from the vanishing of the determinant, detA=0, of the stability matrix

(7) $$A=\left(\begin{array}{ccc} \frac{\partial^{2}f}{\partial \phi^{2}} & \frac{\partial^{2}f}{\partial \phi \partial \psi } & \frac{\partial^{2}f}{\partial \phi \partial \alpha }\\ \frac{\partial^{2}f}{\partial \phi \partial \psi } & \frac{\partial^{2}f}{\partial \psi^{2}} & \frac{\partial^{2}f}{\partial \psi \partial \alpha }\\ \frac{\partial^{2}f}{\partial \phi \partial \alpha } & \frac{\partial^{2}f}{\partial \psi \partial \alpha } & \frac{\partial^{2}f}{\partial \alpha^{2}} \end{array}\right).$$

Points {ϕ,ψ,α} inside the spinodal surface, where A is negative definite, are locally unstable. Tie lines with end points $\left\{\phi_{1},\psi_{1},\alpha_{1}\right\}$ and $\left\{\phi_{2},\psi_{2},\alpha_{2}\right\}$ are determined by the familiar common tangent plane construction [42]

$${\left(\frac{\partial f}{\partial \phi }\right)}_{1}={\left(\frac{\partial f}{\partial \phi }\right)}_{2},\;{\left(\frac{\partial f}{\partial \psi }\right)}_{1}={\left(\frac{\partial f}{\partial \psi }\right)}_{2},\;{\left(\frac{\partial f}{\partial \alpha }\right)}_{1}={\left(\frac{\partial f}{\partial \alpha }\right)}_{2},$$

(8) $$f_{2}-f_{1}=\left(\phi_{1}-\phi_{2}\right){\left(\frac{\partial f}{\partial \phi }\right)}_{1}\;\;+\left(\psi_{1}-\psi_{2}\right){\left(\frac{\partial f}{\partial \psi }\right)}_{1}\;\;+\left(\alpha_{1}-\alpha_{2}\right){\left(\frac{\partial f}{\partial \alpha }\right)}_{1},$$

where we have introduced the abbreviations $f_{1}=f\left(\phi_{1},\psi_{1},\alpha_{1}\right)$, $f_{2}=f\left(\phi_{2},\psi_{2},\alpha_{2}\right)$, ${\left(\partial f/\partial \phi \right)}_{1}={\left(\partial f/\partial \phi \right)}_{\phi_{1},\psi_{1},\alpha_{1}}$ and analogously for ${\left(\partial f/\partial \psi \right)}_{1}$, ${\left(\partial f/\partial \alpha \right)}_{1}$, ${\left(\partial f/\partial \phi \right)}_{2}$, ${\left(\partial f/\partial \psi \right)}_{2}$, and ${\left(\partial f/\partial \alpha \right)}_{2}$. The existence of three distinct points that satisfy the common tangent plane construction defines three-phase coexistence. We also note that the limit of tie lines with vanishingly small separation between the two coexisting compositions $\left\{\phi_{1},\psi_{1},\alpha_{1}\right\}$ and $\left\{\phi_{2},\psi_{2},\alpha_{2}\right\}$ defines a critical point. Critical points are located on the spinodal surface (as determined by Equation (7)); in addition, they fulfill the operator equation

(9) $${\left(B_{\phi }\frac{\partial }{\partial \phi }+B_{\psi }\frac{\partial }{\partial \psi }+B_{\alpha }\frac{\partial }{\partial \alpha }\right)}^{3}f\left(\phi ,\psi ,\alpha \right)=0,$$

where the $B_{\phi }$, $B_{\psi }$, $B_{\alpha }$ are the cofactors of A along one arbitrarily chosen row or column. For example, taking the middle row implies

(10) $$B_{\phi }=\frac{\partial^{2}f}{\partial \phi \partial \alpha }\frac{\partial^{2}f}{\partial \psi \partial \alpha }-\frac{\partial^{2}f}{\partial \phi \partial \psi }\frac{\partial^{2}f}{\partial \alpha^{2}},\;B_{\psi }=\frac{\partial^{2}f}{\partial \phi^{2}}\frac{\partial^{2}f}{\partial \alpha^{2}}-{\left(\frac{\partial^{2}f}{\partial \phi \partial \alpha }\right)}^{2},\;B_{\alpha }=\frac{\partial^{2}f}{\partial \phi \partial \psi }\frac{\partial^{2}f}{\partial \phi \partial \alpha }-\frac{\partial^{2}f}{\partial \phi^{2}}\frac{\partial^{2}f}{\partial \psi \partial \alpha }.$$

We are not aware of previous approaches to express the critical point condition in the operator form of Equation (9). In Appendix A, we briefly discuss the derivation of Equation (9) and state equivalent criteria that appear elsewhere in the literature [45,46,47].

We finally note that tri-critical points require the merging of two critical points. Mathematically, this can be expressed by the vanishing magnitude of the cross product $|\nabla s_{1}\times \nabla s_{2}|=0$, where ∇={∂/∂ϕ,∂/∂ψ,∂/∂α} denotes the gradient, $s_{1}=s_{1}\left(\phi ,\psi ,\alpha \right)=detA$ the determinant of the stability matrix, and $s_{2}=s_{2}\left(\phi ,\psi ,\alpha \right)$ the left-hand side of Equation (9).

### 2.3. Example for Calculation of Spinodals and Critical Points

**Figure 2:**
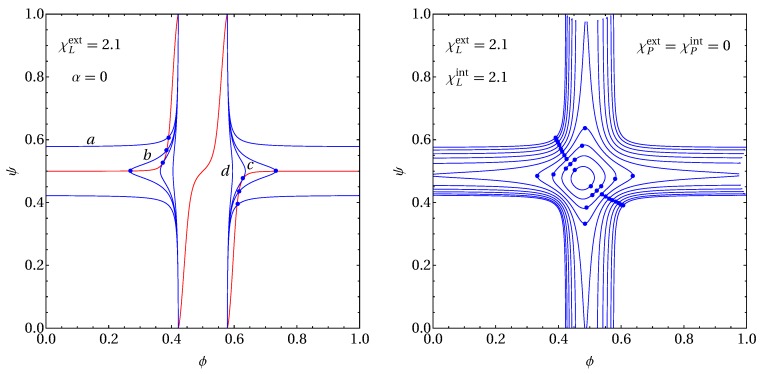
Left diagram: Spinodal lines (curves in blue color) and the solution of Equation (9) (the curve in red color) calculated for α=0, $\chi_{L}^{ext}=2.1$ and the four different choices $\chi_{L}^{int}=2.1$ (spinodal labeled “*a*”), $\chi_{L}^{int}=2.045$ (“*b*”), $\chi_{L}^{int}=2.027$ (“*c*”), and $\chi_{L}^{int}=1.95$ (“*d*”). Intersections of the blue and red lines (marked by blue bullets) specify the critical point locations. A tri-critical point is located on curve “c”. Right diagram: Spinodal lines at fixed $\chi_{L}^{ext}=\chi_{L}^{int}=2.1$ and $\chi_{P}^{ext}=\chi_{P}^{int}=0$ for different α, ranging from α=0 (the outermost spinodal) to α=0.045 (the innermost spinodal) in increments of 0.005. Critical points are marked on each spinodal by blue bullets. All results on the left and right diagram are calculated for Λ=0.05.

In Figure 2, we show two examples for the calculation of spinodal surfaces according to detA=0 (with A specified in Equation (7)) and its critical points (Equation (9), with the cofactors specified in Equation (10)).

To this end, we choose f(ϕ,ψ,α) according to Equation (6) with fixed Λ=0.05. Note that the magnitude of Λ can be obtained by dividing the domain mismatch energy by the cross-sectional area per lipid (typically $0.7\;{\mathrm{nm}}^{2}$). However, the domain mismatch energy is not well known. In the Introduction, we referred to the range $0.1-0.2\;k_{B}T/{\mathrm{nm}}^{2}$ estimated by Risselada et al [21] through Molecular Dynamics simulations, but Putzel et al. [29] argued the domain mismatch energy could be an order of magnitude lower. Clearly, our value Λ=0.05 should be regarded as a rough estimate of a quantity that remains poorly understood.

The left diagram of Figure 2 refers to the absence of proteins, α=0. It displays four spinodal lines (curves in blue color labeled “*a*”–“*d*”) and the solution of Equation (9) (the curve in red color), all calculated at fixed $\chi_{L}^{ext}=2.1$ and the four different choices $\chi_{L}^{int}=2.1$ (spinodal labeled “*a*”), $\chi_{L}^{int}=2.045$ (“*b*”), $\chi_{L}^{int}=2.0272$ (“*c*”), and $\chi_{L}^{int}=1.95$ (“*d*”). Note that there is only a single red curve, independent of $\chi_{L}^{int}$ because the third derivatives present in Equation (9) remove the quadratic dependencies of the lipid–lipid interaction strengths. Intersections of the blue and red curves mark the critical point locations. The spinodal marked “*a*” exhibits two critical points, “*b*” four critical points, “*c*” two tri-critical points, and “*d*” zero critical points. The tri-critical points occur at $\chi_{L}^{int}=2.0272$; their locations are {ϕ,ψ}={0.373,0.526} and, as implied by symmetry, {ϕ,ψ}={1−0.373,1−0.526}={0.627,0.474}.

The right diagram of Figure 2 shows spinodal lines for fixed $\chi_{L}^{ext}=\chi_{L}^{int}=2.1$ and $\chi_{P}^{ext}=\chi_{P}^{int}=0$. The ten different spinodals correspond to different protein mole fractions α, ranging from α=0 (the outermost spinodal) to α=0.045 (the innermost spinodal) in increments of 0.005. Critical points are marked on each spinodal by blue bullets. Clearly, there are two tri-critical points between α=0.040 and α=0.045. Their locations are {ϕ,ψ,α}={0.4376,0.5223,0.04014} and, as required by symmetry, {ϕ,ψ,α}={0.5223,0.4376,0.04014}. The examples presented in Figure 2 will serve as useful reference in the Results section below.

### 2.4. Numerical Calculation of Coexisting Phases

In order to compute phase diagrams, we need to determine the location of coexisting phases. To this end, it is convenient to minimize the composite thermodynamic free energy

(11) $$f_{th}=\sum_{i=1}^{4}\theta_{i}f\left(\phi_{i},\psi_{i},\alpha_{i}\right),$$

of a potentially phase-separated membrane. In Equation (11), we allow for up to four coexisting phases, labeled i=1,2,3,4, with compositions $\left\{\phi_{i},\psi_{i},\alpha_{i}\right\}$ and area fractions $\theta_{i}$. Of the 16 variables $\theta_{i}$, $\phi_{i}$, $\psi_{i}$, $\alpha_{i}$ only 12 are independent because there are four constraints that express the conservation of (1) the total number of lipids and proteins, (2) the number of lipids of type A in the external layer, (3) the number of lipids of type A in the internal layer, and (4) the number of proteins in the membrane. The four constraints read:

(12) $$1=\sum_{i=1}^{4}\theta_{i},\;\phi =\sum_{i=1}^{4}\theta_{i}\phi_{i},\;\psi =\sum_{i=1}^{4}\theta_{i}\psi_{i},\;\alpha =\sum_{i=1}^{4}\theta_{i}\alpha_{i}.$$

Hence, the minimization of $f_{th}=f_{th}\left(\phi ,\psi ,\alpha \right)$ according to Equation (11) with respect to 12 independent variables and fixed interaction parameters $\chi_{L}^{ext}$, $\chi_{L}^{int}$, $\chi_{P}^{ext}$, $\chi_{P}^{int}$, Λ completely specifies the phase behavior and thus can be used to compute all coexisting phases that correspond to a given point {ϕ,ψ,α} in the phase diagram. We point out that the accurate calculation of complete three-dimensional phase diagrams as function of all parameters and their meaningful visualization is a formidable task beyond the scope of the present work. Instead, we focus on a few examples that illustrate the role of transmembrane proteins for domain registration across the bilayer.

### 2.5. Free Energy of a Lipid Membrane That Contains Peripheral Proteins

**Figure 3:**
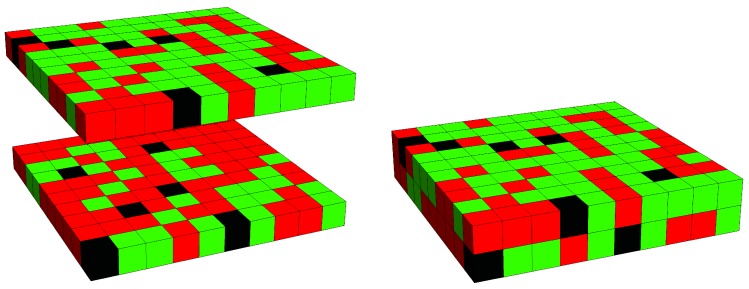
Lattice model for a mixed lipid bilayer that contains “peripheral” proteins. The snapshot shows the two membrane leaflets separated (left diagram) and merged into a bilayer (right diagram). Positions of lipids of type A (green), lipids of type B (red), and peripheral proteins (black) are all uncorrelated across the lattice. While both leaflets may have different lipid compositions, they contain the same number of peripheral proteins. The displayed snapshot corresponds to a square lattice (z=4) of size M=10×10 and mole fractions ϕ=0.61 (the upper leaflet), ψ=0.30 (the lower leaflet), and α=0.06.

The expected mechanism of how transmembrane proteins couple domains across a lipid bilayer is a structural one, based on the ability of the proteins to protrude into (and interact with) both the external and internal membrane leaflets. To assess how effective this mechanism is, we compare transmembrane proteins with “peripheral” proteins, where we obtain 2P peripheral proteins by cutting each of the *P* transmembrane proteins in the middle. The two peripheral proteins that result from one single transmembrane protein are able to independently relocate in their host leaflet; see the illustration in Figure 3.

Two modifications of the free energy are associated with transitioning from transmembrane to peripheral proteins. The first is an increase in the number of available states due to the presence of twice as many proteins

(13) $$\Omega =\frac{M!}{P!\;A_{ext}!\;B_{ext}!}\;\frac{M!}{P!\;A_{int}!\;B_{int}!},$$

which will give rise to the additional free energy contribution αlnα+(1−α)ln(1−α) in the free energy per lattice site. The second is a modification of the inter-leaflet coupling term due to the presence of lipid–protein interactions across the bilayer. To deduce the latter, we first introduce the presence of inter-leaflet interaction strengths between lipid A and protein (${\tilde{\omega }}_{AP}$), between lipid B and protein (${\tilde{\omega }}_{BP}$), and between protein and protein (${\tilde{\omega }}_{PP}$). These interaction strengths are present in addition to the inter-leaflet interaction strengths of lipid A with lipid A (${\tilde{\omega }}_{AA}$), of lipid B with lipid B (${\tilde{\omega }}_{BB}$), and of lipid A with lipid B (${\tilde{\omega }}_{AB}$) as introduced in Equation (4). Recalling that we operate on the level of the random mixing approximation, we recognize that the total inter-leaflet interactions are given by $U_{coupl}/\left(Mk_{B}T\right)=\Lambda {\left(\phi -\psi \right)}^{2}$, where the coupling constant Λ, defined in Equation (4), is independent of ${\tilde{\omega }}_{AP}$, ${\tilde{\omega }}_{BP}$, and ${\tilde{\omega }}_{PP}$. The fact that the inter-leaflet interactions of the peripheral proteins are irrelevant is an immediate consequence of employing the random mixing approximation and the conservation of the number of proteins in the external and internal leaflets. As a consequence, the coupling term in Equation (6) for transmembrane proteins, $\Lambda \left(1-\alpha \right){\left(\phi -\psi \right)}^{2}$, must be replaced by a coupling term $\Lambda {\left(\phi -\psi \right)}^{2}$ for peripheral proteins. Hence, we can write for the mean-field free energy per lattice site in the presence of peripheral proteins

(14) $$\begin{array}{ccc} \tilde{f}\left(\phi ,\psi ,\alpha \right) & = & f\left(\phi ,\psi ,\alpha \right)+\alpha \ln\alpha +\left(1-\alpha \right)\ln\left(1-\alpha \right)+\Lambda \alpha {\left(\phi -\psi \right)}^{2}\\ & = & 2\alpha \ln\alpha +\phi \ln\phi +\psi \ln\psi +(1-\alpha -\phi )\ln(1-\alpha -\phi )+(1-\alpha -\psi )\ln(1-\alpha -\psi )\\ & + & \chi_{L}^{ext}\phi \left(1-\alpha -\phi \right)+\chi_{L}^{int}\psi \left(1-\alpha -\psi \right)+\Lambda {\left(\phi -\psi \right)}^{2}+\chi_{P}^{ext}\alpha \phi +\chi_{P}^{int}\alpha \psi , \end{array}$$

where f(ϕ,ψ,α) has been inserted from Equation (6).

In summary, separating all transmembrane proteins in the membrane into pairs of peripheral proteins that reside in opposite leaflets corresponds to adding the mixing entropy term αlnα+(1−α)ln(1−α) and an inter-leaflet-interaction contribution $\Lambda \alpha {\left(\phi -\psi \right)}^{2}$. We expect the latter to be small because we assume α<1 throughout this work.

### 2.6. A Simple Example for Phase Stability in the Presence of Transmembrane versus Peripheral Proteins

In order to most clearly illustrate the different influence of transmembrane versus peripheral proteins on the phase behavior, we consider the specific situation $\chi_{L}^{int}=\chi_{L}^{ext}=\chi_{P}^{int}=\chi_{P}^{ext}=\chi$, where the proteins interact the same with lipids of type A as lipids of type B interact with lipids of type A. The motivation behind this choice is to eliminate the difference between lipid B and peripheral protein, rendering each leaflet effectively a binary system. We obtain an especially simple free energy expression by demanding identical compositions of the two leaflets, ϕ=ψ. This removes the inter-leaflet coupling term and thus reduces the free energy difference between a membrane with transmembrane proteins and peripheral proteins to the mixing term αlnα+(1−α)ln(1−α).

More specifically, for the peripheral protein, this leads to the free energy $\tilde{f}\left(\phi ,\phi ,\alpha \right)=2\left[\alpha \ln\alpha +\phi \ln\phi +(1-\alpha -\phi )\ln(1-\alpha -\phi )+\chi \phi (1-\phi )\right]$, which amounts to two identical contributions from the two symmetric leaflets. According to Equations (7) and (9), the critical point is located at

(15) $$\chi =2,\;\phi =\frac{1}{2}.$$

Phase separation can only be observed for χ>2, independent of α. Indeed, α is merely a dummy variable because there is no difference anymore between lipid B and peripheral protein: they only differ by their name.

We contrast this to the presence of transmembrane proteins, for which the free energy amounts to $f\left(\phi ,\phi ,\alpha \right)=\alpha \ln\alpha -\left(1-\alpha \right)\ln\left(1-\alpha \right)+2\left[\phi \ln\phi +(1-\alpha -\phi )\ln(1-\alpha -\phi )+\chi \phi (1-\phi )\right]$. Here, the critical point is

(16) $$\chi =2\left(1-\alpha \right),\;\phi =\frac{1}{2},$$

implying that phase separation can already be observed for χ<2 if proteins are present. Let us focus on the case ϕ=1/2 because that always passes exactly through the critical point as α is varied. We can choose α from its minimal value α=0 to its maximal value α=1−ϕ=1/2. In the former case, the membrane consists of 50% lipid A and 50% lipid B, which produces a critical nonideality parameter χ=2. In the latter case, 50% lipid A and 50% transmembrane protein are present, implying a critical nonideality parameter χ=1. Because every transmembrane protein interacts with two leaflets, the lipid–protein interaction strength is effectively doubled. This implies a reduction of the critical nonideality parameter from χ=2 to χ=1. In the intermediate case, for 0<α<1/2, the critical point is predicted by Equation (16) to decrease linearly with increasing mole fraction of transmembrane proteins.

The presence of lipid–protein interactions is crucial for the ability of the transmembrane proteins to facilitate phase separation. If we repeat the same calculation as in the preceding paragraph, yet with $\chi_{L}^{int}=\chi_{L}^{ext}=\chi_{L}$ and $\chi_{P}^{int}=\chi_{P}^{ext}=0$, we obtain the same critical point

(17) $$\chi =\frac{2}{1-\alpha },\;\phi =\frac{1-\alpha }{2},$$

irrespective of the proteins being transmembrane or peripheral. Here, the proteins do not exhibit any interactions with the lipid; they merely dilute the two-component lipid mixture, and they do so in the same way for transmembrane and peripheral proteins. This elevates the critical value for χ and thus suppresses phase separation.

## 3. Results

### 3.1. Phase Behavior in the Absence of Proteins

**Figure 4:**
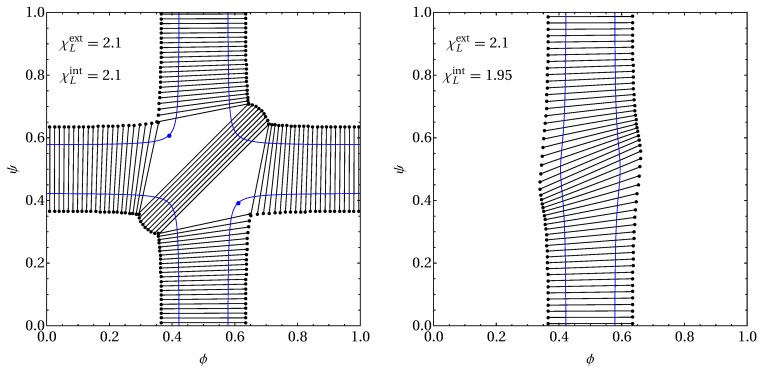
Phase diagrams in the absence of proteins, α=0. The blue lines mark the spinodal. Tie lines (black solid lines) are terminated by black bullets. The left diagram is calculated for $\chi_{L}^{ext}=\chi_{L}^{int}=2.1$ and the right diagram for $\chi_{L}^{ext}=2.1$ and $\chi_{L}^{int}=1.95$. The coupling parameter is Λ=0.05 in both diagrams. The left diagram exhibits two three-phase regions that each hide one critical point, marked by a blue bullet.

We start our analysis by recalling the previously analyzed case α=0, where no proteins are present in the membrane [24,27]. To this end, Figure 4 displays two phase diagrams, both calculated for α=0 and fixed Λ=0.05. They show spinodal lines in blue color with the location of critical points (if present), marked as blue bullets. They also show tie lines as straight solid lines in black, with the two coexisting phases indicated by black bullets at the two ends of each tie line. Regions enclosed by three connected tie lines (present in the left diagram) exhibit three-phase coexistence. The left diagram was calculated for $\chi_{L}^{ext}=\chi_{L}^{int}=2.1$ and the right diagram for $\chi_{L}^{ext}=2.1$ and $\chi_{L}^{int}=1.95$.

We first discuss the left diagram, which can be viewed as a specific example for a class of systems with $\chi_{L}^{ext}=\chi_{L}^{int}=\chi_{L}$. The existence of three-phase coexistence requires a sufficiently small coupling constant $\Lambda <\Lambda^{★}$ with [27]

(18) $$\Lambda^{★}=\frac{3}{2}\;\chi_{L}\;\frac{\chi_{L}-2}{2\chi_{L}-3}.$$

For $\Lambda >\Lambda^{★}$ and $\Lambda <\Lambda^{★}$, the maximum number of coexisting phases is two and three, respectively. The former may be referred to as the strong-coupling regime. At $\Lambda =\Lambda^{★}$, the phase diagram contains two tri-critical points $\phi =\phi^{★}$ and $\psi =\psi^{★}=1-\phi^{★}$ with

(19) $$\phi^{★}=\frac{1}{2}\left(1\mp \sqrt{1-\frac{2}{\chi_{L}}}\right),\;\psi^{★}=\frac{1}{2}\left(1\pm \sqrt{1-\frac{2}{\chi_{L}}}\right).$$

For $\chi_{L}=2.1$, the critical coupling parameter amounts to $\Lambda^{★}=0.2625$, which is well above the value Λ=0.05 used everywhere in the present work. The corresponding locations of the tri-critical points are $\left\{\phi^{★},\psi^{★}\right\}=\left\{0.391,0.609\right\}$ and $\left\{\phi^{★},\psi^{★}\right\}=\left\{0.609,0.391\right\}$. The left phase diagram in Figure 4 makes a number of notable predictions. First, at the boundary of the phase diagram (ϕ=0, ϕ=1, ψ=0, or ψ=1), a two-component leaflet is coupled to a single-component leaflet. The single-component leaflet tends to suppress phase separation in the two-component leaflet. For example, ψ=0 leads to spinodal points ϕ defined by the quadratic equation $2\phi \left(1-\phi \right)=1/\left(\chi_{L}-\Lambda \right)$, and thus an effectively smaller non-ideality parameter $\chi_{L}^{eff}=\chi_{L}-\Lambda$. Second, for ϕ=ψ, the membrane is symmetric and the inter-leaflet coupling term vanishes. The phase behavior is then unaffected by Λ and is determined solely by the non-ideality parameter $\chi_{L}$. That is, the spinodal points for ϕ=ψ are defined by the quadratic equation $2\phi \left(1-\phi \right)=1/\chi_{L}$. Third, the presence of the two three-phase regions for a lipid layer with intermediate asymmetry (where |ϕ−ψ| is neither very small nor large) reflects the competition between inter-leaflet coupling and the tendencies of each leaflet to phase separate. Indeed, two of the three coexisting phases exhibit the same compositional difference, whereas the remaining third phase (which has the largest compositional difference) serves as host for the “non-matching” lipids. We finally note that the critical points (marked by the blue bullets) are inside the three-phase coexistence regions, which render them irrelevant for the thermodynamically observed phase behavior.

Next, we discuss the right diagram of Figure 4. The parameters $\chi_{L}^{ext}=2.1$ and $\chi_{L}^{int}=1.95$ imply that the external but not the internal leaflet tends to phase separate on its own. Hence, phase separation is completely suppressed at small and large ϕ when the outer leaflet resides outside its binodal region. We still observe two-phase coexistence regions, but no three-phase coexistence. Even a different choice of Λ>0 will not give rise to three-phase coexistence. Instead, when Λ grows (starting from Λ=0.05 at fixed $\chi_{L}^{ext}=2.1$ and $\chi_{L}^{int}=1.95$), the spinodal detaches from the borders of the phase diagram (that happens at Λ=0.1, with two critical points appearing at {ϕ,ψ}={0.5,0} and {ϕ,ψ}={0.5,1}) and then forms an increasingly more circular shape. In the limit Λ→∞, the spinodal is a circle of radius 0.079 centered at ϕ=ψ=0.5, with the two critical points {0.556,0.444} and {0.444,0.556} attached. The tilt of the tie lines in the right diagram of Figure 4 is a consequence of the inter-leaflet coupling. The observed positive slope of the tie lines emerges from Λ>0 and the ensuing tendency to minimize the local compositional difference across the bilayer. Any non-vanishing tilt implies distinct compositions of the two coexisting phases in both leaflets. Hence, phase separation in the external leaflet induces phase separation in the internal leaflet. The maximal degree of this “enslaved” phase separation in the inner leaflet is adopted for the tie line that passes the point ϕ=ψ=1/2.

How the two phase diagrams in Figure 4 transform into each other upon decreasing $\chi_{L}^{int}$ from 2.1 to 1.95 is revealed by the spinodals shown in the left diagram of Figure 2: a tri-critical point exists at $\chi_{L}^{int}=2.027$, which separates the presence of three-phase regions (for $\chi_{L}^{int}>2.027$) from its absence (for $\chi_{L}^{int}<2.027$). It is thus interesting to note that for, say, $\chi_{L}^{ext}>2.1$, $\chi_{L}^{int}>2.02$, and Λ=0.05, there exists no three-phase coexistence despite the presence of a non-vanishing inter-leaflet coupling parameter and despite the tendency of both leaflets to phase separate.

### 3.2. Phase Behavior in the Presence of Proteins

Here, we investigate the influence of transmembrane proteins on the phase behavior and compare it with peripheral proteins. For transmembrane proteins, we use f(ϕ,ψ,α) according to Equation (6) and for peripheral proteins $\tilde{f}\left(\phi ,\psi ,\alpha \right)$ according to Equation (14).

**Figure 5:**
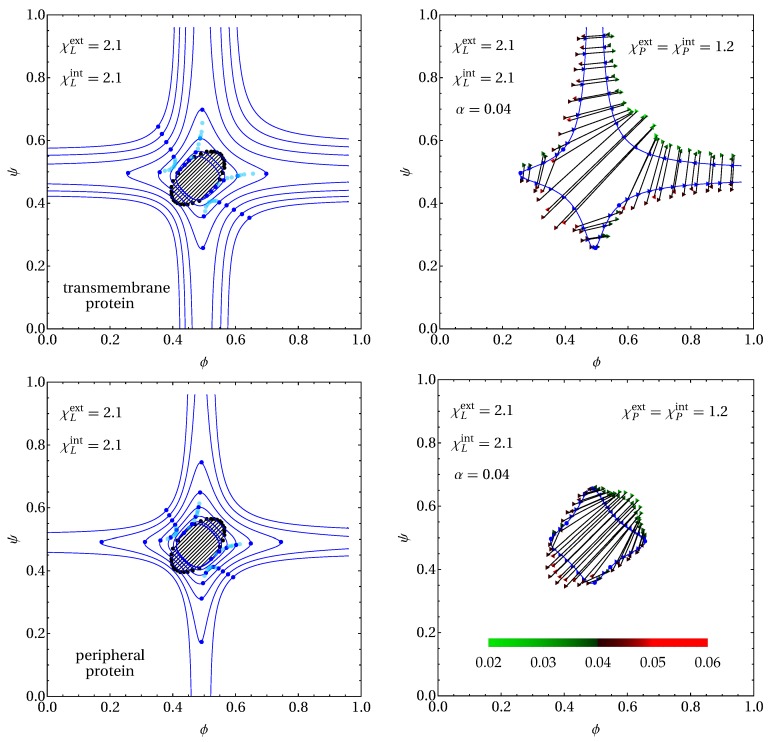
Sections of {ϕ,ψ,α} phase diagrams at α=0.04 for transmembrane proteins (top diagrams) and peripheral proteins (bottom diagrams), with $\chi_{L}^{ext}=\chi_{L}^{int}=2.1$ in both leaflets and Λ=0.05. *Left diagrams*: Spinodal lines (solid lines in blue color) with the locations of the associated critical points (blue bullets), calculated for $\chi_{P}=0$ (the innermost spinodal) and changing in increments of 0.3 until reaching $\chi_{P}=2.1$ (the outermost spinodal). The light-blue bullets mark additional critical points at selected positions without the corresponding spinodal lines being displayed. The tie lines shown (black lines) correspond to the innermost spinodal. Because of $\chi_{P}=0$ for that spinodal, all coexisting phases have a protein mole fraction of 0.04. We have thus marked the two ends of each tie line by black bullets. *Right diagrams*: Spinodal from the corresponding left diagram for $\chi_{P}=1.2$ with a number of selected tie lines, calculated at positions on the spinodal marked by blue triangles. The α-value of the two coexisting phases (at the end of each tie line) are color-coded according to the legend in the bottom right diagram.

Consider first the symmetric case with $\chi_{L}^{ext}=\chi_{L}^{int}=\chi_{L}=2.1$, $\chi_{P}^{ext}=\chi_{P}^{int}=\chi_{P}$, and α=0.04. We initially discuss the left two diagrams of Figure 5. They only differ in the protein type: transmembrane for the top left diagram and peripheral for the bottom left diagram. Each diagram shows eight spinodals (displayed in blue color) with the locations of the associated critical points (blue bullets), calculated for $\chi_{P}=0$ (the innermost spinodal) and changing in increments of 0.3 until reaching $\chi_{P}=2.1$ (the outermost spinodal). The light-blue bullets mark additional critical points without the corresponding spinodal lines being displayed. The innermost spinodal in the upper left diagram, calculated for $\chi_{P}=0$ and α=0.04, has already been displayed in the right diagram of Figure 2. We recall that two sets of three critical points, residing in close vicinity to each other, are located on that innermost spinodal. We have calculated a number of tie lines for the innermost spinodal and added them to the phase diagram (black lines): clearly, the phase diagram exhibits two three-phase regions, but their small size prevents them from being visible, given our choice of tie line positions. Note that, for $\chi_{P}=0$, all coexisting phases have the same protein mole fraction, α=0.04, thus preserving the two-dimensional nature of the phase diagram.

Starting from the innermost spinodal in the upper left diagram of Figure 5 (calculated for $\chi_{P}=0$ and α=0.04), upon slightly increasing $\chi_{P}$, the three critical points merge into a single one. Indeed, the second innermost spinodal has only two single critical points left, and so does the third one (which is calculated for $\chi_{P}=0.6$). Immediately after that, for $\chi_{P}$ slightly larger than 0.6, two tri-critical points appear and then give rise to a total of four additional critical points. To visualize this, we have added multiple light-blue bullets that mark critical point locations between the two spinodals for $\chi_{P}=0.6$ and $\chi_{P}=0.9$. Hence, the next spinodal, calculated for $\chi_{P}=0.9$, contains two sets of three critical points. Two of the six critical points have moved outside the phase diagram boundaries for $\chi_{P}=1.2$, and four of the six critical points have moved outside the phase diagram boundaries for all subsequent spinodals (the three outermost spinodals, calculated for $\chi_{P}=1.5$, $\chi_{P}=1.8$, and $\chi_{P}=2.1$).

The lower left diagram of Figure 5 exhibits a similar scenario as the upper left diagram with two differences. First, the innermost spinodal (which has $\chi_{P}=0$) is very similar in size and shape. All differences result entirely from the different coupling terms (that is, $\left(1-\alpha \right)\Lambda {\left(\phi -\psi \right)}^{2}$ for transmembrane proteins versus $\Lambda {\left(\phi -\psi \right)}^{2}$ for peripheral proteins) but not from the different mixing entropies of the proteins. However, the innermost spinodal in the lower left diagram exhibits only two (instead of six) critical points. That is, the phase diagram for $\chi_{P}=0$ and α=0.04 exhibits three phase regions for transmembrane proteins, but not for peripheral proteins. As in the upper diagram, we have added a number of tie lines to the innermost spinodal; here, no three-phase region is present in the phase diagram. Second, the spinodals for the peripheral proteins enclose smaller regions than the corresponding spinodals for transmembrane proteins. The latter is a major finding of the present work. It quantifies the ability of the transmembrane proteins—even when present at small mole fractions—to induce membrane phase separation, whereas, for equivalent peripheral proteins, the membrane remains uniform. In addition, transmembrane proteins tend to induce or widen three-phase coexistence regions. For example, all tie lines displayed in the left two diagrams of Figure 5 encode for two-phase coexistence where the two phases have the same degree of inter-leaflet mismatch (|ϕ−ψ| is the same in both phases). A third phase, one with larger inter-leaflet mismatch, does not form because of the prohibitively large inter-leaflet domain-coupling energy. However, transmembrane proteins are able to couple mismatching domains structurally, thus counteracting the unfavorable inter-leaflet domain-coupling mechanism.

The outermost spinodals in the upper and lower left diagrams of Figure 5 refer to $\chi_{P}=2.1$, where the interactions of the transmembrane proteins with the A-lipids are the same as the interactions of the B-lipids with the A-lipids (that is, $\chi_{L}=\chi_{P}=2.1$). Peripheral proteins then behave the same as B-lipids, reducing each leaflet effectively to a binary mixture of A-lipids with the union of indistinguishable B-lipids and proteins. Hence, all differences in the phase diagram arise from the presence (for transmembrane proteins) or absence (for peripheral proteins) of the inter-leaflet connectiveness between the proteins segments, without being further affected by the different lipid–lipid and lipid–protein interactions within each leaflet. We have discussed this for the special case of a symmetric membrane (ϕ=ψ) in Section 2.6. The outermost spinodals in the upper and lower left diagrams of Figure 5 clearly demonstrate the widening of the region where the membrane is unstable due to the inter-leaflet connectiveness of the transmembrane proteins.

We have selected one particular spinodal from the upper and lower left diagrams of Figure 5—the fourth one counted from outside, corresponding to $\chi_{P}=1.2$—and calculated a number of tie lines; the results are shown in the two right diagrams of Figure 5. Note that the phase diagrams are three-dimensional, with tie lines and three-phase regions extending out of the plane of the displayed {ϕ,ψ,α=0.04} section of the phase diagram. Hence, unlike in Figure 4, each unstable point {ϕ,ψ} has its own individual tie line (or three-phase region). This makes a meaningful visualization of the phase diagram more challenging. On the two right diagrams of Figure 5, we have simply selected a few points (marked by triangles) located on the spinodal (the solid blue lines with the critical points marked as blue bullets) and calculated the corresponding phase behavior. In all cases, we found two-phase coexistence as characterized by tie lines. The α-values of the end points of the tie lines are color-coded, with green hues for α<0.04 and red hues for α>0.04; see the legend on the bottom right diagram of Figure 5. From the effective lipid A–protein interactions terms in the free energy, $\chi_{P}^{ext}\alpha \phi +\chi_{P}^{int}\alpha \psi$, it follows that proteins preferentially reside in a phase rich in lipid B (that is, small ϕ and small ψ), given that $\chi_{P}^{ext}>0$ and $\chi_{P}^{int}>0$. This has two consequences in the phase diagrams. First, coexisting phases with smaller ϕ and ψ values tend to have larger protein content (i.e., red triangles on the left bottom end and green triangles on the right top end of each tie line). In addition, second, phase separation tends to become more pronounced in regions of larger ϕ and ψ. Regarding the latter, compare, for example, the cases ϕ=0 and ϕ=1−α=0.96 for transmembrane proteins (the upper right diagram in Figure 5). Clearly, there is no phase separation for ϕ=0, but there is phase separation for ϕ=096. Comparing the free energy in Equation (6) shows the presence of the additional term $\chi_{P}^{int}\alpha \left(1-\alpha \right)$ for ϕ=1. This term is thus responsible for the additional destabilization of the membrane. The physical origin of the difference is that, for ϕ=0, the upper leaflet contains only lipid type B, whereas, for ϕ=1, the upper leaflet contains only lipid type A. There is no preferential interaction of the proteins with lipid B, but a tendency for segregation when the protein resides in a matrix of lipid A. Hence, we observe a stronger tendency for phase separation when ϕ=1 as compared to ϕ=0.

**Figure 6:**
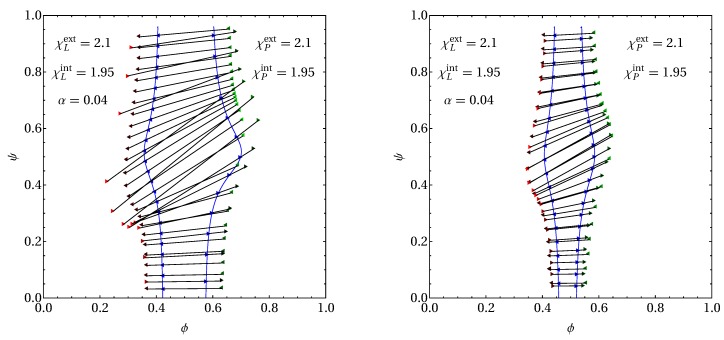
Sections of {ϕ,ψ,α} phase diagrams at α=0.04 for transmembrane proteins (left diagram) and peripheral proteins (right diagram), calculated for $\chi_{L}^{ext}=\chi_{P}^{ext}=2.1$, $\chi_{L}^{int}=\chi_{P}^{int}=1.95$, and Λ=0.05. Both diagrams show a number of selected tie lines, calculated at positions on the spinodal marked by blue triangles. The α-values of the two coexisting phases (at the end of each tie line) are color-coded according to the legend in the bottom right diagram of Figure 5.

Similar considerations are also valid for membranes with asymmetric interactions, $\chi_{L}^{ext}\neq \chi_{L}^{int}$ or $\chi_{P}^{ext}\neq \chi_{P}^{int}$. We exemplify this in Figure 6 for a membrane with $\chi_{L}^{ext}=\chi_{P}^{ext}=2.1$ and $\chi_{L}^{int}=\chi_{P}^{int}=1.95$. As in the two right diagrams of Figure 5, we show a {ϕ,ψ,α=0.04} section of the phase diagram with the spinodal line and a selected number of tie lines displayed. The left and right diagrams in Figure 6 correspond to transmembrane and peripheral proteins, respectively. In fact, the only difference when comparing the upper right diagram of Figure 5 with the left diagram of Figure 6 (for transmembrane proteins) and the lower right diagram of Figure 5 with the right diagram of Figure 6 (for peripheral proteins) is the parameter change from $\chi_{L}^{int}=\chi_{P}^{int}=2.1$ to $\chi_{L}^{int}=\chi_{P}^{int}=1.95$. Hence, because the latter is below the critical point, we no longer observe phase transitions in the small and large ϕ-regions of the phase diagram. By comparing the phase diagram in the right diagram of Figure 4 with those in Figure 6, we directly observe the influence of adding transmembrane or peripheral proteins of mole fraction α=0.04 and interaction strengths $\chi_{P}^{ext}=2.1$ and $\chi_{P}^{int}=1.95$. We also note that the spinodal lines in Figure 6 reproduce the small and large ψ-regions of those in Figure 5 for $\chi_{P}=2.1$ (the outermost spinodals in the two left diagrams of Figure 5). As a result of decreasing $\chi_{P}^{int}$ from 2.1 to 1.95, no critical points are present anymore and phase separation always produces exactly two coexisting phases. Most importantly and similar to our discussion of Figure 5, transmembrane proteins produce substantially larger unstable regions in the phase diagram as compared to peripheral proteins.

We emphasize that the phase diagrams shown in Figure 5 and Figure 6 only contain limited information—spinodals and tie lines at a few selected positions of a {ϕ,ψ,α=0.04}—section. Complete phase diagrams contain information about all coexisting phases at every point {ϕ,ψ,α} in the three-dimensional phase diagram. Visualizing full phase diagrams in a meaningful way and analyzing them comprehensively for the set, $\chi_{L}^{int}$, $\chi_{L}^{ext}$, $\chi_{P}^{int}$, $\chi_{P}^{ext}$, Λ, of interaction parameters is a work in progress.

## 4. Conclusions

Our work is a first step into the direction of analyzing the interplay between “thermodynamic” and “structural” coupling of domains across a lipid membrane. “Thermodynamic” coupling results from the inter-leaflet interactions of the lipids, whereas the coupling becomes “structural” for transmembrane proteins (or other membrane-spanning components such as bolalipids) that stretch across the entire lipid bilayer. We have considered a mean-field, lattice-based model with nearest neighbor pair interactions. This type of model is highly simplistic and in many ways oversimplifies a protein-containing lipid membrane. However, it captures the principal aspect of introducing membrane-spanning molecules (which we refer to as transmembrane proteins) into a lipid bilayer and its influence on the membrane phase behavior. We find that this influence depends on the interaction strength of the transmembrane proteins with the lipids in the two leaflets. Weakly interacting proteins suppress phase separation by merely diluting the lipids. If the lipid–protein interaction strength resembles that among the lipids (which leads to domain formation in the first place), then transmembrane proteins are indeed able to couple domains and enhance or even induce their formation. Our present work may trigger a number of extensions. Perhaps one of the most interesting is related to the ability of immobilized pinning sites to limit the growth of lipid domains [48], which is one among a number of mechanisms [49,50] that have been suggested to rationalize the nanoscopic size of membrane rafts. Cytoskeletal coupling of the inner leaflet of a plasma membrane creates pinning sites, but it is the outer leaflet that has a high propensity to phase separate.

We emphasize the simplicity of our model. For example, all energy penalties due to hydrophobic mismatch (either between the lipids among each other or between the lipids and proteins) are lumped into effective interactions’ parameters. Hence, our model is not capable of making predictions of how domain coupling depends on lipid chain length or on the hydrophobic protein thickness. Moreover, we represent lipids by the sites of two coupled two-dimensional lattices, thus ignoring any molecular specificities such as head group size, degree of lipid chain unsaturation, etc. The special role of cholesterol does not enter explicitly into our model, neither do membrane bending [51], line tension effects of domains [52,53], protein-mediated coupling of domains to its surroundings [54,55,56], or any multi-body interactions. Finally, we treat our lattice model on the mean-field level, which ignores any type of correlations between interacting membrane constituents [57]. However, even with all these approximations, our simple model leads to a surprisingly rich phase behavior. 

## Appendix A. Thermodynamic Criterion for Critical Point

Equation (9) (together with Equation (10)) defines the location of critical points on the spinodal surface. It can be derived starting from the tangent plane construction in Equation (8) by expressing the two coexisting compositions as $\phi_{1}=\phi +▵\phi$, $\psi_{1}=\psi +▵\psi$, $\alpha_{1}=\alpha +▵\alpha$, $\phi_{2}=\phi -▵\phi$, $\psi_{2}=\psi -▵\psi$, $\alpha_{2}=\alpha -▵\alpha$. Upon carrying out a series expansion up to first order in x={▵ϕ,▵ψ,▵α}, we can express the three equal-slope equations in the first line of Equation (8) as Ax=0, thus reproducing the equation detA=0 that defines the spinodal. With that, the first non-vanishing term in the expansion with respect to ▵ϕ, ▵ψ, ▵α of the second line of Equation (8) is of third order. The two eigenvectors of x allow us to express two of its components by the remaining one; for example, ▵ϕ=▵ϕ(▵ψ) and ▵α=▵α(▵ψ). Inserting these into the third-order expansion of the second line of Equation (8) yields

(A1) $$\begin{array}{ccc} 0 & = & B_{\phi }^{3}\frac{\partial^{3}f}{\partial \phi^{3}}+B_{\psi }^{3}\frac{\partial^{3}f}{\partial \psi^{3}}+B_{\alpha }^{3}\frac{\partial^{3}f}{\partial \alpha^{3}}+6B_{\phi }B_{\psi }B_{\alpha }\frac{\partial^{3}f}{\partial \phi \partial \psi \partial \alpha }\\ & + & B_{\phi }^{2}B_{\psi }\frac{\partial^{3}f}{\partial \phi^{2}\partial \psi }+B_{\phi }^{2}B_{\alpha }\frac{\partial^{3}f}{\partial \phi^{2}\partial \alpha }+B_{\phi }B_{\psi }^{2}\frac{\partial^{3}f}{\partial \phi \partial^{2}\psi }+B_{\psi }^{2}B_{\alpha }\frac{\partial^{3}f}{\partial^{2}\psi \partial \alpha }+B_{\phi }B_{\alpha }^{2}\frac{\partial^{3}f}{\partial \phi \partial^{2}\alpha }+B_{\psi }B_{\alpha }^{2}\frac{\partial^{3}f}{\partial \psi \partial^{2}\alpha }, \end{array}$$

where our choice of the independent variable in expressing the two eigenvectors determines which row (or, equivalently, column) the cofactors $B_{\phi }$, $B_{\psi }$, $B_{\alpha }$ refer to. Equation (A1) is equivalent to Equation (9).

We have not observed Equation (9) to be stated previously in the literature. However, multiple equivalent ways to express Equation (9) are well known; see the discussions by Akasaka [46] and by Bell and Jäger [47]. Among those is a set of equations that goes back to Heidemann and Khalil [45] and results from a third-order stability analysis of a Taylor series expansion of the Helmholtz free energy about the equilibrium state. Another method involves the vanishing of the determinant detB=0 where the matrix B is produced from matrix A (see Equation (7)) by replacing any one of its rows by the row vector {∂(detA)/∂ϕ,∂(detA)/∂ψ,∂(detA)/∂α} [58].
